# Brain Renin–Angiotensin System: From Physiology to Pathology in Neuronal Complications Induced by SARS-CoV-2

**DOI:** 10.1155/2023/8883492

**Published:** 2023-08-04

**Authors:** Shamseddin Ahmadi, Shiler Khaledi

**Affiliations:** Department of Biological Science, Faculty of Science, University of Kurdistan, Sanandaj, Iran

## Abstract

Angiotensin-converting enzyme 2 (ACE2), a key enzyme in the renin–angiotensin system (RAS), is expressed in various tissues and organs, including the central nervous system (CNS). The spike protein of severe acute respiratory syndrome coronavirus 2 (SARS-CoV-2), the virus responsible for coronavirus disease-2019 (COVID-19), binds to ACE2, which raises concerns about the potential for viral infection in the CNS. There are numerous reports suggesting a link between SARS-CoV-2 infection and neurological manifestations. This study aimed to present an updated review of the role of brain RAS components, especially ACE2, in neurological complications induced by SARS-CoV-2 infection. Several routes of SARS-CoV-2 entry into the brain have been proposed. Because an anosmia condition appeared broadly in COVID-19 patients, the olfactory nerve route was suggested as an early pathway for SARS-CoV-2 entry into the brain. In addition, a hematogenous route via disintegrations in the blood–brain barrier following an increase in systemic cytokine and chemokine levels and retrograde axonal transport, especially via the vagus nerve innervating lungs, have been described. Common nonspecific neurological symptoms in COVID-19 patients are myalgia, headache, anosmia, and dysgeusia. However, more severe outcomes include cerebrovascular diseases, cognitive impairment, anxiety, encephalopathy, and stroke. Alterations in brain RAS components such as angiotensin II (Ang II) and ACE2 mediate neurological manifestations of SARS-CoV-2 infection, at least in part. Downregulation of ACE2 due to SARS-CoV-2 infection, followed by an increase in Ang II levels, leads to hyperinflammation and oxidative stress, which in turn accelerates neurodegeneration in the brain. Furthermore, ACE2 downregulation in the hypothalamus induces stress and anxiety responses by increasing corticotropin-releasing hormone. SARS-CoV-2 infection may also dysregulate the CNS neurotransmission, leading to neurological complications observed in severe cases of COVID-19. It can be concluded that the neurological manifestations of COVID-19 may be partially associated with changes in brain RAS components.

## 1. Introduction

The classical circulating renin–angiotensin system (RAS) regulate blood pressure, body fluid volume, electrolyte balance, and cardiac and renal functions [1]. Angiotensin-converting enzyme (ACE) is one of the key components of the RAS, which is responsible for converting angiotensin I (Ang I) to Ang II with a strong vasoconstrictor role [2]. However, the activity of ACE in the RAS is counterbalanced by another enzyme, ACE2, which converts Ang II to its metabolite, Ang-1–7, with opposite effects to Ang II. ACE2 is expressed in different tissues and organs, including the central nervous system (CNS) and the peripheral nervous system (PNS) [3, 4]. In addition, finding other RAS components in the CNS has verified the existence of a local brain RAS with multiple functions in health and disease [5, 6].

Severe acute respiratory syndrome coronavirus 2 (SARS-CoV-2), the virus responsible for coronavirus disease-2019 (COVID-19), invades various cells, including brain cells, by binding to ACE2 on cell membranes, thereby supporting the neurotropism of the virus [7, 8]. Multiple nonspecific symptoms, such as hyposmia, anosmia, dysgeusia, headache, and dizziness, have been reported in COVID-19. In addition, numerous reports support the existence of more intensive neurological complications following SARS-CoV-2 infection, including cerebrovascular diseases, cognitive impairment, anxiety, encephalopathy, and stroke. This study aimed to review the physiological functions of both the circulating and brain RAS components and to summarize the latest findings on neuronal complications due to SARS-CoV-2 infection, highlighting the role of the brain RAS components in pathological mechanisms underlying neuronal complications associated with COVID-19.

## 2. Classical and Nonclassical RAS

Independent classical and nonclassical RAS systems are involved in the regulation of blood pressure, body fluid volume, electrolyte balance, and cardiac and renal functions [9, 10]. Renin, angiotensinogen, and ACE are the main components of the classical circulating RAS [10]. Renin is a protease that is synthesized from a precursor known as prorenin in juxtaglomerular cells located in the wall of the afferent glomerular arteriole in the glomeruli of the kidney and is released into circulation under low blood pressure conditions. Angiotensinogen is a plasma protein that is mostly produced by hepatocytes and cleaved into a decapeptide product known as Ang I in circulation by the enzymatic action of renin [10–12]. The inactive decapeptide Ang I is converted to an active vasoconstrictor octapeptide known as Ang II by the action of ACE, a central component of the RAS. Upon binding to its specific receptors, Ang II triggers a broad range of biological actions that influences almost every system, including the vasculature, heart, kidney, immune system, and brain [10].

Angiotensinogen is also synthesized in other tissues, including the heart, blood vessels, kidney, lung, adipose tissue, adrenal gland, ovaries, large intestine, stomach, spleen, brain, and spinal cord. Therefore, there are different nonclassical local RAS in the body that operates independently of or in parallel with the classical RAS [9]. In addition, nonclassical RAS in different organs can affect the functions of classical RAS. For instance, the brain RAS affects systemic blood pressure by influencing sympathetic tone [13]. In addition to various physiological functions, a growing body of evidence suggests potential roles for the brain RAS in the pathogenesis of blood–brain barrier (BBB) permeability, neurodegenerative diseases, and cognitive impairments [14, 15].

### 2.1. Angiotensin Receptors Mediate Functions of RAS

There are two major classes of G-protein coupled receptors for Ang II: Ang II subtype-1 receptor (AT1R) and Ang II subtype-2 receptor (AT2R). Ang II has the highest relative affinity for AT1R. In particular, Ang II binds to AT1R on smooth muscle cells in the blood vessels and causes vasoconstriction, leading to an increase in blood pressure. Ang II also stimulates the adrenal cortex to secrete aldosterone, which in turn stimulates the distal tubules and collecting ducts of nephrons to increase sodium reabsorption and potassium secretion, thereby maintaining the sodium and water balance as well as potassium homeostasis [16]. Considering the role of RAS in salt and water retention, ACE inhibitors and AT1R antagonists are significant strategies for treating hypertension [17]. In addition to its vasoconstrictor role, many adverse biological effects, such as myocardial hypertrophy, interstitial fibrosis, endothelial dysfunction, inflammation, obesity-associated hypertension, oxidative stress, and increased coagulation, are mediated by Ang II acting on AT1R [18]. In contrast, Ang II binding to AT2R opposes the unfavorable actions of Ang II on AT1R, leading to vasodilation, anti-inflammation, antifibrosis, antioxidation, and capillary integrity [19, 20].

### 2.2. ACE and ACE2: From Physiology to Pathology

The *ACE* gene, located on chromosome 17, encodes a transmembrane ectoenzyme. *ACE* mRNA expression has been reported in many organs throughout the body, but its protein expression is predominant in the lung, gastrointestinal tract, kidney, and male and female reproductive glands. ACE is a membrane-bound protein located mainly on the vascular endothelium and epithelial, neuroepithelial, and immune cells. It acts as a dipeptidyl carboxypeptidase that breaks down the last two amino acids from Ang I to produce Ang II [21]. Therefore, ACE, as a key component of RAS, plays a pivotal role in the regulation of blood pressure indirectly via the production of Ang II [22]. A soluble form of ACE has also been reported to be cleaved from membrane-bound ACE, but its physiological roles remain unclear. Furthermore, ACE functions are not limited to the RAS, and it also cleaves the c-terminal residue from other peptides such as bradykinin, dynorphin 1–13, and apelin, which may, in turn, control blood pressure [23].

The gene encoding *ACE2* is located on the X chromosome and was first introduced in 2000 [24]. It encodes an ectoenzyme with an extracellular catalytic domain that hydrolyzes the circulating peptides. ACE2 is a homolog of ACE with approximately 40% identity and 61% sequence similarity. However, despite the sequence similarity between ACE and ACE2, they have different tissue distributions [24]. SARS-CoV-2 uses ACE2 as a cell receptor to invade human cells, prompting new research to investigate the distribution of ACE2 across organs. Li et al. [25] reported the highest levels of ACE2 in the small intestine, testis, kidneys, heart, thyroid, and adipose tissue and the lowest in the blood, spleen, bone marrow, brain, blood vessels, and muscle. In addition, ACE2 showed moderate expression levels in the lungs, colon, liver, bladder, and adrenal gland [25]. ACE2 is present in neurons, endothelial cells, glial cells, and the choroid plexus in the CNS.

**Figure 1:**
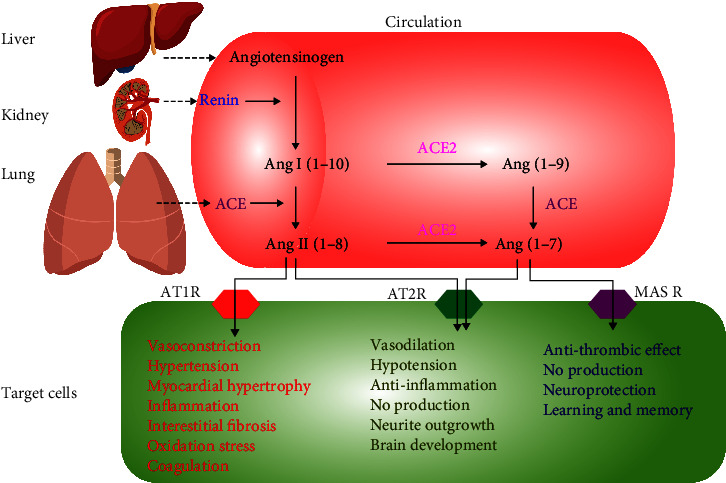
Renin–angiotensin system (RAS) components and functions. Angiotensinogen is a plasma protein that is mostly produced in the liver. Renin is synthesized in juxtaglomerular cells in the kidney glomeruli and converts angiotensinogen to Ang I (1–10). Angiotensin-converting enzyme (ACE) located on apical membranes of endothelial cells in different organs, especially in the lungs, catalyzes the conversion of Ang I to a potent vasoconstrictor Ang II (1–8). ACE2 is also a protease that converts Ang I (1–10) to Ang-(1–9) and Ang II (1–8) to Ang-(1–7). The actions of Ang II on target cells are mediated by two types of receptors, AT1R and AT2R, which lead to opposing effects. Ang-(1–7) also binds to AT2R, and another receptor known as the mitochondrial assembly (MAS) receptor, has positive effects on target cells.

ACE2 is a carboxypeptidase rather than a dipeptidyl carboxypeptidase that removes only one amino acid residue from the C-termini of target proteins. ACE2 acts on both Ang I and Ang II, but it has 400 times more affinity for Ang II [26]. As a result, it produces Ang-(1–9) from Ang I but a much higher amount of Ang-(1–7) from Ang II. Ang-(1–9) is also converted to Ang-(1–7) by ACE [26–28]. Ang-(1–7) via AT2R causes vasodilation and reduces inflammation and oxidative stress responses, which thereby counterbalances the effects of Ang II on AT1R. Ang-(1–7) also exerts some of its biological effects by binding to another G-protein-coupled receptor termed the mitochondrial assembly (MAS) receptor [29]. Ang-(1–7), via binding to the MAS receptor, triggers the production of nitric oxide (NO) and prostaglandin-12, leading to an improvement in blood flow [30, 31], as well as antioxidative and antithrombotic effects and neuroprotection [32]. Therefore, the ACE2/Ang-(1–7)/AT2R and MAS receptor pathways exert protective effects by counterbalancing the ACE/Ang II/AT1R axis [26, 33]. The ACE2 carboxyl-terminal domain is a homolog of a protein known as collectrin, with 48% sequence identity. This domain plays a key role in the absorption of amino acids, especially tryptophan and glycine, in the small intestine and also plays a role in their reabsorption in the kidney by binding to the amino acid transporter BAT1, thereby regulating amino acid levels in the blood. Therefore, ACE2 deficiency is followed by impaired amino acid uptake in the gut leading to malnutrition [34, 35]. Animal models with genetic alterations in ACE2 expression show different complications, including hypertension, metabolic and behavioral dysfunction, deficits in serotonin synthesis, and neurogenesis [36] (Figure 1).

## 3. ACE2 Is a Receptor for SARS-CoV-2 Entry into the Host Cells

SARS-CoV2 invades the host cells via the ACE2 receptor [37]. The distribution of ACE2 in different tissues provides a route for viral infection. Recent studies have demonstrated that the ACE2 receptor is also expressed in the brain, particularly in neurons and glial cells in certain regions such as the brainstem, hypothalamus, and cortex, raising concerns about the neurotropic potential of COVID-19. Experimental studies have also shown the expression of ACE2 in the brain vasculature with the highest density in capillaries found in the olfactory bulb, the paraventricular, supraoptic, and mammillary nuclei of the hypothalamus, the midbrain substantia nigra, and ventral tegmental area, the hindbrain pontine nucleus, the pre-Botzinger complex, and the nucleus; the tractus solitarius. ACE2 is expressed in key components of the BBB, including astrocytes, astrocytic foot processes, pericytes, and endothelial cells in the human and mouse brain [7, 38]. The distribution of ACE2 in the human brain indicates that SARS-CoV-2 infection may cause neurological outcomes in COVID-19 patients.

The envelope spike protein of SARS-CoV-2 is a key structure for ACE2 binding [39]. This protein has 1,273 amino acids and is composed of two subunits: S1 and S2. It also has an S1/S2 cleavage site, and its proteolysis into S1 and S2 subunits by cellular proteases is essential for virus activation. The presence of a four-amino acid sequence inserted between the S1 and S2 domains is different between SARS-CoV-2 and SARS-CoV. This sequence introduces a cleavage site for furin or proprotein convertase subtilisin/kexin type 3, which converts the inactive proprotein into an active spike protein. Preactivation by furin could allow transmembrane serine protease 2 (TMPRSS2) to prim the S1/S2 cleavage site and then process by endosomal cysteine proteases such as cathepsin B and L. Besides, a disintegrin and metalloprotease 17 (ADAM17) also cleaves and processes ACE2, which in turn causes binding of the virus, facilitating TMPRSS2 availability, viral entry by membrane fusion of the virus, and finally developing pathogenesis of the virus [40]. Other studies have also demonstrated that the entry of the virus into target cells depends on other potential receptors, such as CD26 as a cell entry receptor for MERS-CoV and CD147, also known as Basigin [41, 42]. Therefore, the interplay between different factors in the entry of the virus may be responsible for the variability in host susceptibility to SARS-CoV-2 infection [43]. In addition, dysregulation of the RAS cascade in cardiovascular disease, hypertension, and diabetes can also increase susceptibility to SARS-CoV-2 infection [44].

**Figure 2:**
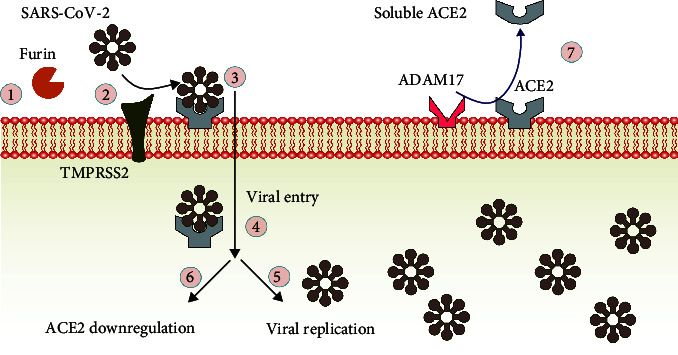
Viral entry into host cells via binding to ACE2. (1) Furin is a proprotein convertase that processes the spike protein of SARS-CoV-2, (2) preparation conditions for the action of TMPRSS2, (3) binding of the virus to ACE2 on target cells, (4) upon viral entry via endocytosis, (5) viral replication progresses by employing organelles, and (6) endocytosis of ACE2 from the cell membrane into the cells causes downregulation of ACE2, which is followed by an increase in Ang II and subsequent adverse functions. Furthermore, ADAM17 can catalyze membrane-bound ACE2 to soluble conversion, and its plasma levels can be considered a biomarker for the prognosis of different diseases, including COVID-19.

The binding of SARS-CoV-2 to ACE2 increasingly leads to the downregulation of the ACE2/SARS-CoV-2 complex, followed by an increase in Ang II, which may, in turn, inhibit ACE activity by negative feedback. ACE inhibition prevents the conversion of active bradykinin into inactive fragments. As a result, excessive bradykinin induces leaky blood vessels and the accumulation of fluid in the lungs [45]. Therefore, ACE2 acts directly as a receptor for viral entry and is indirectly involved in the pathology of SARS-CoV-2 in the lungs via bradykinin. A different pattern of ACE and ACE2 expression in different individuals in the population explains why some people are more susceptible to severe or fatal symptoms [46, 47]. The soluble forms of both ACE and ACE2 are generated from a cleavage secretion process by transmembrane metalloproteases such as ADAM17, which are enzymatically active in body fluids. Elevated levels of circulating soluble ACE2 can also be considered a biomarker for the prognosis of various diseases, including COVID-19 [4, 48]. Thus, there may be a balance between tissue-bound and soluble forms of ACE and ACE2 to maintain their physiological functions (Figure 2).

## 4. Brain RAS Components: From Physiology to Pathology

For the first time, Ganten et al. [49] introduced a renin-like enzyme in the brain independent of the kidney and plasma renin. ACE2 and Ang II are also expressed in various brain cells and regions, including neurons, astrocytes, cerebral circumventricular organs, and cerebrovascular endothelial cells [50]. Several studies have shown the existence of ACE and angiotensinogen in astrocytes and renin in neurons and astrocytes [6]. Further, it has been revealed that Ang II binds to AT1R and AT2R in neurons, astrocytes, oligodendrocytes, and microglial cells [6, 51]. These data confirm the existence of a local RAS in the brain that is involved in water and sodium homeostasis and the regulation of cerebral blood flow [52]. However, chronic activation of the brain RAS and subsequently elevated levels of Ang II also induce pathophysiological conditions such as neuroinflammation, oxidative stress, and age-related diseases [53]. It has also been shown that neurodegenerative diseases, including Alzheimer's disease (AD), Parkinson's disease (PD), multiple sclerosis (MS), and amyotrophic lateral sclerosis, are partially related to dysfunction of the brain RAS [54, 55]. Moreover, decreased cognitive function due to Ang II-mediated AT1R activation has also been reported [54]. Ang III, a metabolite of Ang II that is found in the brain and cerebrospinal fluid, also acts on AT1R. Ang-IV acts on AT4R in addition to its effects on AT1R and AT2R. The activation of AT4R stimulates the release of acetylcholine from the hippocampus, affecting learning and memory processes in rats [56]. The RAS also cross-talks with the kallikrein–kinin system in the brain. ACE converts bradykinin into its inactive form, thereby preventing its vasoconstrictive role. Bradykinin and its receptors B1R and B2R are involved in CNS diseases. Although kinin B1R is associated with neurodegenerative diseases, B2R plays a protective role [57, 58]. However, the exact mechanisms underlying neurodegeneration caused by the brain RAS remain elusive. Together, these data support the involvement of brain RAS components in physiological functions and neuropathological conditions in COVID-19.

## 5. Different Ways for SARS-CoV-2 Entry into the CNS

Direct and indirect routes of SARS-CoV-2 entry into the CNS have been proposed. The virus may infect the PNS either directly via nerve endings in peripheral tissues and reach the CNS through retrograde axonal transport or indirectly via a hematogenous route [59]. Studies have shown that the epithelium of the oral and nasal cavities, as well as the respiratory tract, expresses ACE2 [60], supporting the hypothesis that SARS-CoV-2 may reach the brain through some cranial nerves. Many investigations have also reported that sensory neurons of the olfactory system express ACE2, which may be a route for viral entry into the olfactory bulb and subsequently other parts of the CNS. In addition, infected nerve endings in other peripheral nerves and retrograde transport of SARS-CoV-2 toward the CNS cannot be excluded [61, 62].

Furthermore, tight junctions between vascular endothelial cells and end fits of astrocytes that ensheath capillaries of the CNS create the BBB. Considering the BBB and blood-cerebrospinal fluid barrier as important obstacles against substance entrance into the brain tissue, SARS-CoV-2 entrance into the brain via the intact BBB seems debatable. BBB disruption appears to be the main route for virus entry into the brain and its subsequent neurological implications [63]. The virus can cross the BBB and is distributed throughout the brain because of ACE2 expression in the vascular endothelium, pericytes, and astrocytes of the circumventricular organ, along with parenchymal neurons and glial cells [64, 65].

BBB leakage is the main cause of age-dependent neuropathology [66]. Astrocytes are in direct contact with endothelial cells that maintain BBB integrity, affecting BBB permeability by releasing biomolecules such as Endothelin1, inflammatory cytokines, and NO [67]. The expression of several genes related to inflammation in endothelial cells, such as vascular cell adhesion molecule 1 and intercellular adhesion molecule 1, is upregulated due to SARS-CoV-2, influencing BBB integrity and enabling immune cell migration into the brain parenchyma [62]. An increase in matrix metallopeptidase 9 and the breakdown of collagen in the basement membranes also increase BBB permeability, which contributes to many CNS disorders [68]. Consistent with the high expression of ACE2 and TMPRSS2 in choroid plexus (ChP) epithelial cells [69], in vivo and in vitro findings have revealed that SARS-CoV-2 does not readily infect neurons but infects choroid plexus epithelial cells [63]. Finally, the presence of blood proteins in the CSF of more than 40% of COVID-19 patients supports the idea that BBB leakage is a route for viral entry into the ChP [70].

Compared with other areas of the brain, BBB permeability in the subventricular zone develops an apoptotic effect on neuronal stem cells and diminishes the number of migrating cells to the olfactory bulb, which may, in turn, cause a reduction in the sense of smell [61]. However, some evidence suggests that this route is different from that in humans [62, 71]. On the contrary, Brann et al. [60] also reported that human and mouse olfactory sensory neurons do not express ACE2 and TMPRSS2, which are essential enzymes for virus entry into host cells, suggesting that nonneuronal mechanisms may be involved in anosmia in COVID-19 [61].

**Figure 3:**
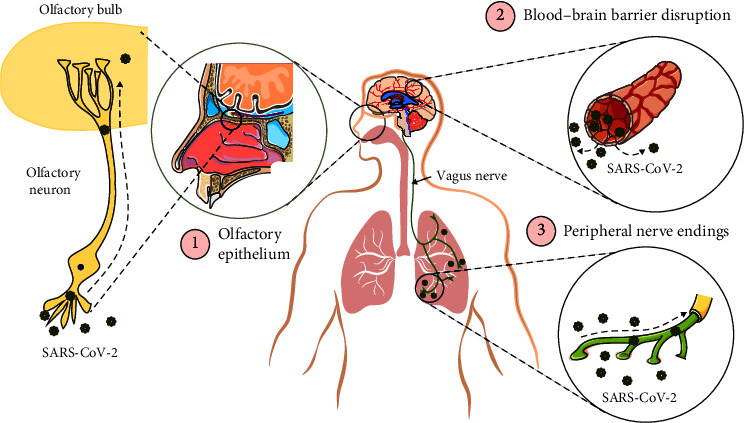
Different routes for SARS-CoV-2 entry into the brain. (1) Olfactory epithelium and sensory olfactory neurons, (2) leakage of the blood–brain barrier (BBB) due to systemic inflammation, and (3) retrograde transport from the vagus nerves innervating the lungs and other visceral organs.

Other investigations have also reported widespread ACE2 expression in brain stem areas involved in the regulation of cardiorespiratory functions [72–74]. Furthermore, ACE2 is present in noncardiovascular areas such as the motor cortex and raphe nucleus, ventricles, and substantia nigra, as well as in brain regions that are related to olfactory processing, including the hypothalamic nuclei, hippocampus, amygdala, and orbitofrontal cortex [75]. Data from animal experiments have demonstrated that hACE2 transgenic mice inoculated intracranially with SARS-CoV display the presence of the virus in the dorsal vagal complex located in the medulla oblongata [76]. Axons of the vagus nerve arise from the dorsal vagal complex and innervate the lung and respiratory tract, implying that this nerve may also serve as a peripheral nerve route for SARS-CoV-2 entry into the brain [77] (Figure 3). One hypothesis suggests that SARS-CoV-2 infects the cardiorespiratory control center in the brainstem, which may ultimately lead to death in patients with severe COVID-19 [78].

## 6. Neurological Manifestations of SARS-CoV-2 Mediated by Brain RAS Components

During the COVID-19 pandemic, it was revealed that the sites of virus infection are not limited to the lungs, and many other organs, such as the heart, kidney, testes, and brain, are affected [79]. Cumulative studies show that COVID-19 patients also exhibit neurological manifestations, including anosmia, ageusia, and severe headache as primary symptoms, and encephalopathy and stroke as secondary consequences [80]. The potential impact of these neurological consequences on morbidity and mortality, especially in severe cases of hospitalized patients with COVID-19, cannot be excluded [81]. In vitro experiments have revealed that SARS-CoV-2 but not SARS-CoV replicates in neuronal U251 cells supporting the potential role of the virus in manifesting neurological symptoms [82].

SARS-CoV-2 infection can induce downregulation of ACE2 that, in turn, increases Ang II levels. Elevated Ang II levels are considered the main cause of the release of reactive oxygen species (ROS) and inflammatory mediators, leading to neurodegeneration in the brain [83]. Direct interaction of the virus with endothelial cells activates the immune cells. ACE2 expression in macrophages also increases inflammatory cytokine and chemokine levels, resulting in lymphocyte depletion and an increased circulating neutrophil ratio [84]. Downregulation of ACE2 in peripheral vessels provokes the activation of the ACE/Ang II/AT1R pathway, which may promote vascular injury. Systemic hyperinflammation related to the innate immune system can also cause endothelial dysfunction in the brain parenchyma, activate CNS microglia, and increase cytokine and chemokine production [84]. In addition to systemic inflammatory lesions in the CNS, direct viral infection of astrocytes, microglia, and macrophage as CNS immune cells increases inflammatory cytokine production, followed by tissue damage [85]. In addition to innate immune responses, an autoimmune reaction may occur via adaptive immune activation in response to the coronavirus infection [86]. Therefore, dysregulated immune responses and high levels of inflammatory cytokines may induce neurological manifestations of COVID-19 [62]. Given the presence of ACE2 in different brain areas, the development of neurological manifestations of COVID-19 is inevitable. Understanding the possible mechanisms of neuroinvasion by the virus can help to identify better treatment strategies and prevent its damaging effects on the CNS and PNS [87].

### 6.1. Brain RAS, SARS-CoV-2, and Hyperinflammation

Toll-like receptors (TLRs) can recognize diverse components of SARS-CoV-2 infection, including viral pathogen-associated molecular patterns and host cell damage-associated molecular patterns, to induce innate immune activation [88]. Therefore, SARS-CoV-2 infection in severe cases is usually accompanied by hyperinflammation and cytokine storm through activation of TLRs on the plasma and endosomal membranes of innate immune cells [89, 90]. Research shows that most COVID-19 patients suffer from a decrease in lymphocytes and an increase in neutrophils, as well as high serum levels of proinflammatory cytokines and chemokines (Table 1).

Furthermore, purinergic receptors on neurons and immune cells are activated by extracellular ATP as a physiologic proinflammatory immune response that initiates cytokine release [96]. It has been revealed that SARS-CoV-2 infection drives cellular ATP release, which in turn activates P2X receptor (P2XR) purinergic signaling and increases immune responses and inflammatory processes [93, 97]. It has been reported that prolonged activation of P2X7Rs following uncontrolled ATP release during SARS-CoV-2 infection causes a cytokine storm and cell death. Therefore, P2X7R antagonists have been proposed to alleviate hyperinflammation and restore normal immune function in COVID-19 patients [98].

In the physiological context, the increased production of Ang-(1–7) by the action of ACE2 inhibits mitogen-activated protein kinase and NF-*κ*B signaling cascade via activation of MAS receptors, resulting in reduced production of inflammatory cytokines. Research has revealed that SARS-CoV-2 infection through the downregulation of ACE2 receptors deteriorates the existing balance between the anti-inflammatory nonclassical RAS axis (ACE2/Ang-(1–7)/MAS receptor) and inflammatory classical RAS pathway (ACE/Ang II/AT1R) in favor of increasing inflammation. Therefore, SARS-CoV-2 infection indirectly triggers a cytokine storm by influencing ACE2 levels [99]. In addition, cytokine storm and inflammation due to SARS-CoV-2 infection may result in dysregulated cerebral neurotransmission and neurological complications observed in patients with severe COVID-19 [87]. Together, hyperinflammation induced by either activation of TLR/purinergic receptors or changes in ACE2 receptors may be a reasonable cause of subsequent neuronal complications in COVID-19 patients [87]. However, some investigators suggest that hyperinflammation, although observed in severe cases of COVID-19, may not be the primary cause of neurological manifestations, and other factors should be considered.

Activation of microglia by Ang II induces upregulation of TNF-*α*, which contributes to dopaminergic neurodegeneration. In addition, *α*-synuclein produced by microglia results in the apoptosis of dopaminergic neurons in the substantia nigra, which is supported by pharmacological treatment with ACE1 and AT1R blockers [100]. Therefore, ACE2 plays a protective role against the development of PD by converting Ang II to Ang-(1–7). Together, SARS-CoV-2 affects brain RAS components, especially the downregulation of ACE2, inducing oxidative stress, which in turn may cause further neurodegeneration and neurological complications.

### 6.2. Brain RAS, SARS-CoV-2, and Oxidative Stress

An imbalance in the reduction-oxidation (redox) system is a hallmark of many metabolic disorders and neurodegenerative diseases [101]. NADPH oxidase (NOX) and mitochondrial transport electron chain are two main sources of endogenous ROS and reactive nitrogen species [102]. Research suggests that SARS-CoV-2 infection can trigger an imbalance in the redox state of host cells and their ability to manage ROS, leading to oxidative stress. This state of oxidative stress can cause severe damage to cellular components, leading to inflammation, vasoconstriction, and neurological dysfunction in COVID-19 patients [103]. Ang II is considered a main factor in the release of ROS and inflammatory mediators, which contribute to neurodegeneration through NOX activation [83]. It has been shown that increased production of ROS in specific areas of the brain, such as the hypothalamus and rostral ventral medulla, followed by oxidative stress, increases the sympathetic tone, resulting in vascular dysfunction, baroreflex sensitivity, and hypertension [104]. However, ACE2 may reduce Ang II-mediated oxidative stress in the brain and prevent autonomic dysfunction. In addition, the presence of ACE2 in the brain regulates cardiovascular function by reducing Ang II levels [104]. It has also been reported that *ACE2* deletion in mice results in age-related oxidative stress in the CNS [104]. However, the exact molecular mechanisms underlying the antioxidant effects of ACE2 remain unknown and require further investigation. ACE2 also promotes vasodilation by catalyzing the conversion of Ang II to Ang-(1–7), which stimulates eNOS followed by NO release, thereby counteracting the pathological effects of Ang II [105]. Therefore, a possible mechanism for the induction of oxidative stress in SARS-CoV-2 infection is partly mediated by ACE2 downregulation and subsequent Ang II/AT1 receptor overactivation.

### 6.3. Brain RAS, SARS-CoV-2, and Cognitive Impairment

There are reports on the effect of COVID-19 on cognitive function [106]. Brain imaging techniques on participants infected with SARS-CoV-2 have revealed reductions in gray matter thickness in some areas of the cortex and white matter, which was accompanied by a cognitive decline [107, 108]. The distribution of ACE2 in different brain regions is not homogeneous. The expression of ACE2 in both neurons and glia in cortical and subcortical regions has been reported [38, 109]. Therefore, COVID-19 patients are vulnerable to neurodegenerative diseases, such as PD and MS, due to the adverse effects of SARS-CoV-2 infection on neurons and glial cells [110, 111]. Many studies have reported a link between hypertension as a risk factor for vascular disease and reduced cognitive function in AD [112]. ACE, on the one hand, is a key enzyme in Ang II-mediated hypertensive pathogenesis, but on the other hand, it is thought to be a favorable enzyme for degrading A*β*, preventing senile plaque formation, and increasing acetylcholine release [112]. Research shows that the ACE2 expression decreases with age. This age-related reduction in ACE2 reduces its ability to counterbalance the functions of Ang II, potentially contributing to the severity of COVID-19 symptoms like hypertension and inflammation induced by Ang II in the elderly population [113]. Alternative research has indicated that ACE2 is increased in individuals with AD, potentially amplifying the pathogenicity of SARS-CoV-2 in such patients [114].

Furthermore, there is a modulatory connection between neurotrophic factors and the brain RAS. The expression of neurotrophin-3 and brain-derived neurotrophic factor (BDNF) is regulated by Ang II [115]. Brain angiotensin peptides can potentially modulate brain function and improve cognition [116]. Activation of AT1R by elevated Ang II due to local RAS activation in the brain plays an important role in vasoconstriction, neuroinflammation, neuronal death, and cognitive impairment [115]. Animal models with ACE2 deficiency exhibit significant impairments in memory and cognition, which can be partly rescued by Ang-(1–7) and AT1R antagonists. It has been postulated that Ang-(1–7) and its receptor may be involved in memory processing in the hippocampus [36]. Hippocampal neuronal stem cells are responsible for producing new neurons in the adult brain. Thus, hippocampal lesions are associated with memory loss and reduced cognition [117]. Reduced tryptophan and serotonin levels in ACE2 knockout mice compared to those in wild-type animals have been shown to reduce hippocampal neurogenesis [36]. Ang II dysregulates memory mediators such as acetylcholine, vascular endothelial growth factor, and *N*-methyl-D-aspartate subtype of glutamate receptors in the brain. Kim et al. [117] have demonstrated that cardiovascular disorder and continuous RAS activation, which are followed by an increased level of Ang II via AT1R, induce apoptotic death in hippocampal neural stem cells, developing memory impairments by ROS formation from a mitochondrial source. This is characterized by altered mitochondrial morphology and function, which may account for hypoxic outcomes in the brain and anaerobic metabolism [117]. Alterations in BDNF expression and increased generation of ROS contribute to the impairment of memory and cognitive functions [36]. These results suggest that Ang-(1–7)/MAS receptors may be potential targets for the treatment of cognitive impairment. Decreasing Ang II activity has been shown to have beneficial effects on the brain, including reduced blood pressure and acetylcholine release [112]. However, the growing concern is the persistence of cognitive disorders for a long time in COVID-19 survivors.

### 6.4. Brain RAS, SARS-CoV-2, and Stroke

COVID-19 has been identified as a risk factor for stroke [118, 119]. It is well established that Ang II constricts the smooth muscles of arteries, raising thrombotic effects by stimulating the plasminogen activator inhibitors PAI1 and PAI2. Conversely, Ang-(1–7) has been shown to have antithrombotic and antihypertensive effects via the MAS receptor by promoting NO production in platelets [32]. ACE2 exerts its beneficial effects in stroke models by shifting the balance between Ang II/AT1R in favor of the Ang-(1–7)-MAS receptor axis to control blood pressure [36]. Inflammatory responses to SARS-CoV-2 and elevated complement levels also evoke thrombosis, and abnormal coagulation cascades [61]. Hypoxia is also considered a major cause of neurological manifestations of COVID-19. Severe lung injuries induce hypoxia and anaerobic metabolism in the mitochondria of cerebral cells, which is the main result of acid production, followed by reduced blood flow, impaired BBB, cerebral edema, ischemia, and severe headache. It can increase the risk of stroke, especially in patients with a history of cardiovascular disease. Due to BBB permeability, secondary inflammation may be the cause of neural symptoms, including encephalopathy in COVID-19 [120].

### 6.5. Brain RAS, SARS-CoV-2, and Anxiety

The impact of the COVID-19 pandemic on the occurrence of major depressive and anxiety disorders has been globally evaluated by the COVID-19 Mental Disorders Collaborators (2021). They reported that the COVID-19 pandemic led to an increase in anxiety disorders [121]. Anxiety is often accompanied by dysregulation of the hypothalamus–pituitary–adrenal axis and increased levels of proopiomelanocortin (POMC) and corticosterone [122]. In addition, different types of stress are associated with sympathetic nerve activity, which in turn, results in increased plasma levels of renin and Ang II. It has been shown that circulating Ang II acts as an important stress hormone by binding to AT1R in the brain. This was corroborated by ameliorating stress disorders by blocking AT1 receptors [123]. Animal models with changes in Ang-(1–7)/MAS receptor activity exhibited changes in anxiety-like behaviors, which were abolished by MAS receptor antagonists, supporting the involvement of Ang-(1–7) in the stress response and anxiety. Alenina and Bader [36] reported that ACE2 overexpression in the hypothalamus inhibits corticotropin-releasing hormone synthesis and consequently decreases POMC and corticosterone release, which in turn suppresses the stress response and anxiety. The above-cited data support this suggestion that ACE2 downregulation following SARS-CoV-2 infection leads to a high level of Ang II, which in turn may induce anxiety in COVID-19 patients.

## 7. Conclusion

Several routes have been identified for SARS-CoV-2 entry into the CNS. There are considerable reports in favor of linking the involvement of brain RAS components in SARS-CoV-2 infection and subsequent neurological complications. The interaction between ACE2 and SARS-CoV-2 leads to the downregulation of ACE2. Therefore, an imbalance between ACE and ACE2 followed by an increased level of Ang II as well as primary and secondary inflammation induced by SARS-CoV-2 infection may be plausible causes of neuronal complications in COVID-19 patients. Headache, dizziness, and confusion are common nonspecific symptoms that have been reported in the initial stages of the illness in most patients with COVID-19. However, elevated inflammatory and oxidative stress markers in severe cases can cause multiple neurological syndromes with a more complex presentation. ACE2 has neuroprotective properties on neuronal cells mostly via the production of Ang-(1–7) from Ang II, which in turn activates MAS receptors. The downregulation of ACE2 due to SARS-CoV-2 infection is associated with a decrease in its neuroprotective capacity in the CNS, making neural cells more susceptible to injury. It can be concluded that SARS-CoV-2 infection increases the possibility of neurodegeneration and neurological disorders by affecting RAS components in the brain. However, further studies are needed to understand the exact relationship between the local brain RAS components and the neurological manifestations of COVID-19.

## Figures and Tables

**Table 1:** Changes in immune cells and inflammatory markers in the severe cases of COVID-19 patients.

Inflammatory markers	Changes	References
Immune cells:		
T cell, B cell, and NK cell	↓	[91, 92]
Leukocyte and neutrophil count	↑	
Neutrophil/lymphocyte ratio	↑	
Cytokines:		
TNF-*α*, IL1-*β*, IL1-R*α*, IL2, IL2-R IL6, IL8, IL10, IL12, IL-18, G-CSF, GM-CSF	↑	[91, 93, 94]
Chemokines:		
CCL2, CCL3, CCL4, CXCL10	↑	[95, 89]

NK cell, natural killer cell; TNF-*α*, tumor necrosis factor-*α*; IL1-*β*, interleukine-1*β*; IL-2R, interleukin 2-receptor; G-CSF, granulocyte colony-stimulating factor; GM-CSF, granulocyte-macrophage colony-stimulating factor; IFN-*γ*, interferon-*γ*; CCL2, C-C motif chemokine ligand-2; CXCL10, CXC motif chemokine ligand-10.

## Data Availability

Data sharing is not applicable to this article as no new data were created or analyzed in this study.
